# An unusual cause of fatal rapid-onset ataxia plus syndrome

**DOI:** 10.1186/s40673-017-0063-9

**Published:** 2017-04-21

**Authors:** Ivan Kmezic, Jan Weinberg, Dan Hauzenberger, Farouk Hashim, Evangelia Kollia, Monika Klimkowska, Inger Nennesmo, Martin Paucar

**Affiliations:** 1 Department of Neurology, Karolinska University Hospital, 141 86 Stockholm, Sweden; 2 Department of Clinical Immunology, Karolinska University Hospital, Stockholm, Sweden; 3 Department of Radiology, Karolinska University Hospital, Stockholm, Sweden; 4 Department of Clinical Pathology and Cytology, Karolinska University Hospital, Stockholm, Sweden; 5 Department of Clinical Neuroscience, Karolinska Institute, Stockholm, Sweden

**Keywords:** PML, Ataxia, JC-virus, Polycythemia vera, Hydroxyurea, FASCIA analysis

## Abstract

**Background:**

Progressive multifocal leukoencephalopathy (PML) is a demyelinating disorder of the central nervous system caused by reactivation of the JC-virus and is in most cases associated with underlying immunosuppression. Acquired immune deficiency syndrome (AIDS) and hematological malignancies are well-known predisposing factors for PML. However, in the past ten years, various pharmacological agents have been associated with increased risk of PML. Based on the phenomenology PML can be divided into the cerebral form and the rare cerebellar form.

**Case presentation:**

Here we describe a man affected by polycythemia vera (PCV) that was treated with hydroxyurea (HU) and developed PML. The initially PML presentation included ataxia as one of the main features. Brain MRI displayed widespread supratentorial and infratentorial lesions. Immunological analysis revealed absence of reactivity to a wide range of antigens. The course of disease was rapidly progressive with fatal outcome - autopsy ruled out leukemic transformation.

**Conclusion:**

The occurrence of PML in PCV patients is very rare and has been reported only once. Movement disorders, such as ataxia, are also less frequent. In the present case the PML was likely multifactorial.

## Background

The association between polycythemia vera (PCV) and progressive multifocal leukoencephalopathy (PML) is very rare and has been reported only once [1]. This previously reported patient with PCV was treated with interferon (IFN)-α2B and thalidomide, but not hydroxyurea. In most cases PML is associated with immunodeficiency [2, 3]; however in very rare occasions it may also afflict immunocompetent patients [4]. The most frequent symptoms are motor weakness, changes in mentation and impaired vision [5], whereas movement disorders are less frequently seen [6].

## Case presentation

**Fig. 1:**
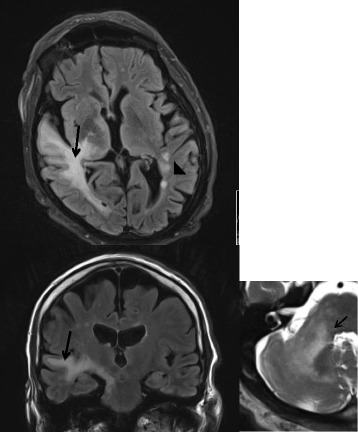
Brain MRI displaying PML features in a PCV patient treated with hydroxyurea. Axial and coronal T1-weighted sections displaying multifocal and confluent supra- and infratentorial lesions without contrast enhancement (*arrows*). There are also supratentorial punctate lesions (indicated by an arrow head). To the right T2-weigthed section displaying lesions in the right cerebellar hemisphere (*arrow*). No restricted diffusion

An 81 year-old man presented with subacute disorientation, mild left-side weakness, impaired balance, apraxia and unilateral spatial neglect in May 2015. His past medical history included arterial hypertension and a stable course of PCV, diagnosed at the age of 66, treated with hydroxyurea (HU), low dose acetylsalicylic acid (ASA) and occasional phlebotomy. Upon admission in May 2015 a brain CT scan revealed a hypodense area in the right temporal lobe initially interpreted as a subacute infarction. Mild anemia was found and HU was discontinued. The patient was referred to a rehabilitation clinic, but his condition deteriorated rapidly in the following days. On readmission left-side weakness, marked contralateral limb ataxia, dysdiadochokinesia, bradykinesia, dysarthria, left homonymous hemianopia and left-sided hemianesthesia were evident. Brain MRI performed at this point displayed multifocal and punctate slightly expansive white matter non-contrast enhanced juxta- and subcortical lesions, predominantly in the right hemisphere. These lesions were more confluent around the right trigonum and within the right temporal lobe and brainstem. There was involvement of the right cerebral peduncle, anterior pons on the right side, right middle cerebellar peduncle and the cerebellum around the dentate nucleus. On diffusion-weighted imaging (DWI) lesions were hyperintense but without restricted diffusion as the apparent diffusion coefficients (ADC) were not decreased in the area (Fig. 1). EEG-recording revealed non-epileptiform abnormalities such as slow wave activities over right hemisphere and maximum over right temporal region.

**Fig. 2:**
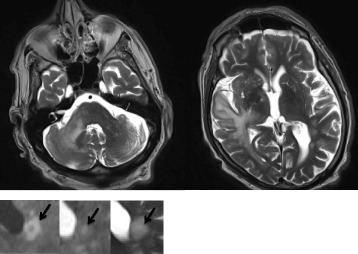
Brain MRI on day 38 of admission in a male with underlying PCV who developed PML. Axial T2-weighted sections display increased number of widespread and confluent supra- and infratentorial lesions. There were several “ring-shaped” lesions with hyperintensive periphery on DWI, increased central signal and isointense periphery on ADC. On T2-weighted images the lesions were globally hyperintense

A lumbar puncture was performed and yielded normal cell count and protein content in the cerebrospinal fluid (CSF). Enterovirus, herpes simplex 1 and 2, varicella zoster and CMV, were ruled out in the CSF by means of PCR and ELISA analysis. Serology for Lyme’s disease and syphilis in the CSF was also negative. Onconeural antibodies were not detected in blood. A centrifugated CSF sample was analyzed with fluorescence-activated cell sorting (FACS) which identified only few T-cells. Neurofilament light protein (NfL) was markedly elevated (16500 ng/L; reference interval <1850). Analysis for JC-virus yielded 7 400 copies/mL and tests for BK-virus, HIV, and HTLV-1 tests were negative. A second MRI of the brain three weeks after admission displayed an increased number of widespread supra- and infratentorial lesions (Fig. 2). Additional laboratory investigation at this point revealed mild lymphopenia with decreased amounts of all lymphocyte fractions but not NK-cells. A flow-cytometric assay of specific cell-mediated immune response in activated whole blood (FASCIA) analysis revealed poor response to mitogens and specific antigens as shown in table (Table 1).

**Table 1:** FASCIA analysis

Analysis	Results	Reference interval
CD 4 PWM	106	233 – 2189 c/μL
CD 8 PWM	5	50 – 549 c/μL
CD 19 PWM	17	42 – 741 c/μL
CD 4 ConA	113	620 – 3800 c/μL
CD 8 ConA	1	180 – 1757 c/μL
CD 4 Influenza	0	19 – 1050 c/μL
CD 4 PPD	0	11 – 2022 c/μL
CD 4 Candida	0	51 – 1014 c/μL

Flow-cytometric assay of specific cell-mediated immune response in activated whole blood (FASCIA-analysis) revealed response absence of reactivity to a wide range of antigens. Response to lectins (pokeweed mitogen and concanavalin A) was moderately mitigated, whereas response to influenza antigen, purified protein derivative antigens and Candida antigen was completely absent

**Fig. 3:**
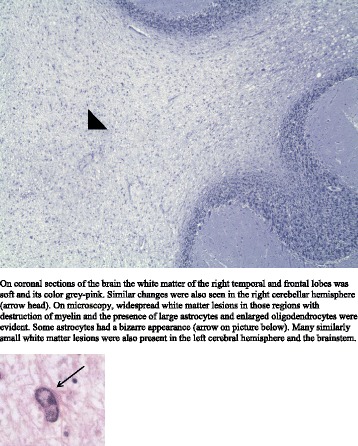
Histopathological findings. On coronal sections of the brain the white matter of the right temporal and frontal lobes was soft and its color grey-pink. Similar changes were also seen in the right cerebellar hemisphere (*arrow head*). On microscopy, widespread white matter lesions in those regions with destruction of myelin and the presence of large astrocytes and enlarged oligodendrocytes were evident. Some astrocytes had a bizarre appearance (*arrow* on picture below). Many similarly small white matter lesions were also present in the left cerebral hemisphere and the brainstem

Different therapeutic options for PML were considered [7], but due to the rapid clinical deterioration treatment was withheld. The patient developed manifest left-sided hemiplegia, horizontal nystagmus, and verbal unresponsiveness. At this point his condition had worsened and the subject became unable to swallow, he contracted recurrent pneumonias and required a percutaneous endoscopic gastrostomy. During the later course of disease he remained in an unresponsive state until he died of pneumonia. Total disease duration in this case was two months. Post mortem studies of the bone marrow demonstrated a picture consistent with PCV but no signs of leukemic transformation. Histopathological analysis of the brain confirmed progressive multifocal leukoencephalopathy (PML) (Fig. 3).

## Discussion and Conclusions

PML is often a fatal disease as in this described case. It can be divided into the cerebral form which is more common and the cerebellar from. The latter can also affect the brainstem [8, 9]. Infratentorial PML is rare, and so far there are only 30 case reports of infratentorial PML in the PubMed database [10]. In the presented case the initial symptom was hemiplegia, but only within a few days a focal movement disorder, i.e. limb ataxia, became one of the main features of the patient’s condition.

Very few sporadic cases of PML have been described and reported in individuals without obvious immunosuppression or immunosuppressive risk factors [6, 8]. The vast majority of PML infections affect immunosuppressed patients. A CD4 count below 200 cells/μL has been identified as a major risk factor for PML in deeply immunosuppressed patients with AIDS [11]. On the other hand, deleterious effects of hydroxyurea on CD4 lymphocytes have been described in children [12], but not in adults. In addition, myeloid malignancy such as PCV is not perceived as an immunosuppressive state. However, the natural process of immunological senescence involves both quantitative shifts and decreased functional capacity of various lymphocyte population subsets. Poor response of lymphocytes to mitogens can be seen in patients with immunological dysfunction or in patients with immunosuppressive treatments. The FASCIA results described here should therefore be interpreted in the context of a mild lymphopenia. In the described case, old age, myeloid malignancy and chemotherapy were likely the three factors that could have potentially contributed to JC-virus reactivation and development of a fatal disease. It is however difficult to pinpoint a single cause of PML in this case. Nevertheless, awareness of the potential consequences of such a constellation is important. PML should be considered as a differential diagnosis, especially in a PCV patient with rapidly progressive neurological symptoms without obvious immunosuppression or leukemic transformation.

## Abbreviations

AIDS: Acquired immune deficiency syndrome
CNS: Central nervous system
CSF: Cerebrospinal fluid
FASCIA: Flow-cytometric assay of specific cell-mediated immune response in activated whole blood
HU: Hydroxyurea
JCV: John Cunningham virus
PCV: Polycythemia vera
PML: Progressive multifocal leukoencephalopathy
